# Identification of a GTP-bound Rho specific scFv molecular sensor by phage display selection

**DOI:** 10.1186/1472-6750-8-34

**Published:** 2008-03-31

**Authors:** Marine Goffinet, Patrick Chinestra, Isabelle Lajoie-Mazenc, Claire Medale-Giamarchi, Gilles Favre, Jean-Charles Faye

**Affiliations:** 1 NSERM U563, CPTP, "Signalisation cellulaire, GTPase Rho et cancers", F-31052, Toulouse, France; 2 Institut Claudius Regaud, Département de Biologie, F-31052, Toulouse, France; 3 Université Paul Sabatier, Faculté des Sciences Pharmaceutiques, F-31062, Toulouse, France

## Abstract

**Background:**

The Rho GTPases A, B and C proteins, members of the Rho family whose activity is regulated by GDP/GTP cycling, function in many cellular pathways controlling proliferation and have recently been implicated in tumorigenesis. Although overexpression of Rho GTPases has been correlated with tumorigenesis, only their GTP-bound forms are able to activate the signalling pathways implicated in tumorigenesis. Thus, the focus of much recent research has been to identify biological tools capable of quantifying the level of cellular GTP-bound Rho, or determining the subcellular location of activation. However useful, these tools used to study the mechanism of Rho activation still have limitations. The aim of the present work was to employ phage display to identify a conformationally-specific single chain fragment variable (scFv) that recognizes the active, GTP-bound, form of Rho GTPases and is able to discriminate it from the inactive, GDP-bound, Rho in endogenous settings.

**Results:**

After five rounds of phage selection using a constitutively activated mutant of RhoB (RhoBQ63L), three scFvs (A8, C1 and D11) were selected for subsequent analysis. Further biochemical characterization was pursued for the single clone, C1, exhibiting an scFv structure. C1 was selective for the GTP-bound form of RhoA, RhoB, as well as RhoC, and failed to recognize GTP-loaded Rac1 or Cdc42, two other members of the Rho family. To enhance its production, soluble C1 was expressed in fusion with the N-terminal domain of phage protein pIII (scFv C1-N1N2), it appeared specifically associated with GTP-loaded recombinant RhoA and RhoB via immunoprecipitation, and endogenous activated Rho in HeLa cells as determined by immunofluorescence.

**Conclusion:**

We identified an antibody, C1-N1N2, specific for the GTP-bound form of RhoB from a phage library, and confirmed its specificity towards GTP-bound RhoA and RhoC, as well as RhoB. The success of C1-N1N2 in discriminating activated Rho in immunofluorescence studies implies that this new tool, in collaboration with currently used RhoA and B antibodies, has the potential to analyze Rho activation in cell function and tumor development.

## Background

The Rho GTPases proteins are members of a large superfamily of regulatory proteins whose activities are controlled by regulated GDP/GTP cycling. To date, a total of 22 Rho family members have been suggested by the data available from the human genome sequence project. The Rho GTPases can be divided into six groups: Rho (RhoA, RhoB, RhoC), Rac (Rac1, Rac2, Rac3, RhoG), Cdc42 (Cdc42, TC10, TCL, Chp/Wrch-2, Wrch-1), Rnd (Rnd1, Rnd2, Rnd3/RhoE), RhoBTB (RhoBTB1 and RhoBTB2) and Miro (Miro-1 and Miro-2). Additional members, RhoD, Rif and TTF/RhoH, do not fall into any of these subfamilies [1].

Rho GTPases control a wide variety of signal transduction pathways regulating many fundamental processes of cell biology, such as organization of the actin cytoskeleton [2], gene expression, cell proliferation and survival [3]. Most Rho proteins cycle between an active GTP-bound state and an inactive GDP-bound state. Binding to GTP is promoted by Rho guanine nucleotide exchange factors (Rho-GEFs), and GTP hydrolysis is catalysed by Rho GTPase-activating proteins (Rho-GAPs). Rho-GDP dissociation inhibitors (Rho-GDIs) stabilize the GDP-bound form of Rho proteins.

Rho proteins are also implicated in participating in several steps of tumor progression and development of metastasis [4,5]. Activated Rho proteins cooperate strongly with oncogenes Ras and Raf in focus-formation assays, but either fail to independently induce transformation or else exhibit weak transforming activity [6-9]. They function in cell cycle regulation by the modulation of cyclin D1 [10] and by their involvement in endocytic traffic [11,12], such as in regulation of epidermal growth factor receptor [13]. Furthermore, Lacal *et al *have shown that Rho GTPases are directly involved in signalling pathways that trigger either proliferation or cell death [14]. Moreover, expression of activated Rac protects against Ras-induced apoptosis [15].

These studies linking Rho proteins to many aspects of cellular proliferation are further extended by the study by Gomez del Pulgar, which revealed that several human tumors contained aberrant expression and activation of Rho GTPases [16]. Elevated expression of RhoA and RhoC was found in breast, lung, ovarian, gastric, and bladder cancers. The involvement of RhoA in testicular human tumors was demonstrated by increased RhoA mRNA levels in relation to tumour grade [17]. Overexpression of the *rhoC *gene in adenocarcinoma of pancreas correlated with poorer prognosis of patients [18], whereas RhoB expression is lost in several tumors [19]. Moreover, unlike Ras, no mutated, constitutively active forms of Rho proteins in tumors have thus far been identified [20], apart from one report linking hyperactive Rac3 with highly proliferative human breast cancer cells and tumor tissues [21]. Whatever the level of gene expressions of Rho GTPases and assuming that high level of protein could be associate with higher concentration of activated Rho GTPase, the knowledge of accurate variations of the Rho activation under treatment would be a significant progress in the understanding of the biological role of Rho in oncogenesis.

To further develop understanding of Rho activation, we identified a conformation-specific scFv against the active form of RhoA, RhoB and RhoC GTPases. We employed a phage display approach, which has previously demonstrated successful results in generating precise sensors of variations in molecular conformation [22]. To this end, we utilized the Q63L mutant [23] of RhoB (locked in the GTP binding structure), and expressed this mutant as a GST fusion protein in *E. coli *and used the purified recombinant protein as a target ligand for selection of recombinant scFv. Using the Griffin.1 library [24], one clone (C1) was selected for its ability to discriminate between the activated versus the unactivated form of RhoB, as well as RhoA and RhoC, but not other Rho family members. The selected C1 scFv was fused to a fragment of phage coat protein pIII gene to enhance its production, expressed in *E. coli*, and its specificity confirmed. Finally, its ability to detect endogenous activated Rho *in vivo *in immunofluorescence studies is shown.

## Results and Discussion

### Identification of RhoBQ63L specific interacting phages

**Figure 1:**
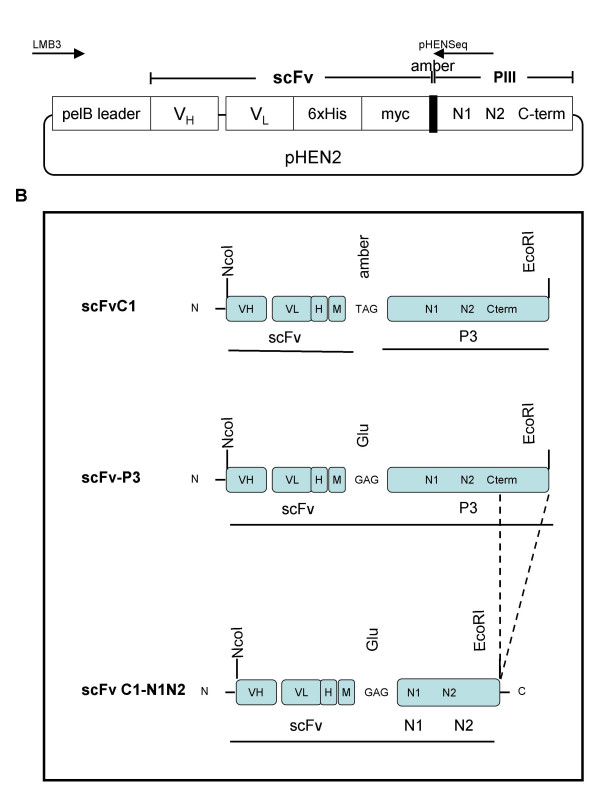
**pHEN2 vector and construction of scFv C1-N1N2**. **A: **Schematic representation of the pHEN2 phagemid vector (Griffin 1. library). *pelB leader*: signal peptide sequence of bacterial pectate lyase that mediates secretion into the periplasmic space; *V*_H _: variable fragment of the heavy chain; *V*_L_: light chain; *6xHis*: 6 histidine-tag; *myc*: myc-tag; *amber: *amber stop codon; *N1, N2, C-term*: portions of the N- and C-term of phage capside protein pIII; *LMB3 *and *pHENSeq*: primers used for sequencing the V_H _and V_L _domain. **B: **Schematic construction of vectors encoding soluble scFv C1 and scFv C1-N1N2. The amber stop codon between the scFv and gene III in pHEN 2 was removed by mutagenesis (middle construct). The C-terminal portion of pIII was removed in the final pHEN C1-N1N2 vector (bottom construct), first by PCR amplification of pHEN C1-pIII, introducing an EcoRI site after N2, and subsequently by cloning the NcoI and EcoRI digested PCR product into the linearized pHEN C1-pIII plasmid at the NotI and EcoRI sites. *H*: 6 histidine-tag; M: myc-tag.

Peptide and protein libraries on filamentous phages, in combination with effective selection strategies, have proven to be a rapid and successful method to identify proteins with specific binding properties. A major advantage of the *in vitro *selection process by phage display is the extensive control of the selection conditions, which, for example, allowed the preservation of the three-dimensional structure of GTP-bound form of Rho. Using the Griffin.1 phage display library, Perez et al identified scFvs that act as molecular conformation sensors by showing selectivity towards the GTP-bound conformation of the Rab6 GTPase [22]. The present work used the same phagemid library containing more than 10^9 ^independent clones. Each clone expresses human recombinant antibodies as a single-chain Fv (scFv), where both *myc, 6xHis *tags, and the pIII protein of M13 are fused to the C-terminus of immunoglobulin V_H_-V_L _fusion (Fig 1).

Due to its ability to decrease the kinetics of GTP hydrolysis, RhoBQ63L is considered as a "GTP-locked" mutant of RhoB. We expressed and purified the GST-tagged RhoBQ63L mutant in Escherichia coli, and used the recombinant protein as target for phage selection. For the first round of selection, we collected phage bound to GST-RhoBQ63L-bound beads. For the subsequent four rounds, selection was a two-part process: we first preblocked phage on GST-beads, and the phage mixture was then loaded on GST-RhoBQ63L beads. During the last round, soluble recombinant wild-type RhoB was added into the phage mixture to specifically select phage that interact only with the active GST-RhoBQ63L form on the beads. Rounds IV and V contained polyclonal phage specific to GST-RhoBQ63L. To increase isolation of positive clones, we initiated two independent screenings from round V: V-1 and V-2. In total, 144 (48 from the V-1 and 96 from the V-2) clones were randomly picked and checked by ELISA, yielding 8 RhoBQ63L selective phages.

### Characterization of the selected single chain antibodies

**Figure 2:**
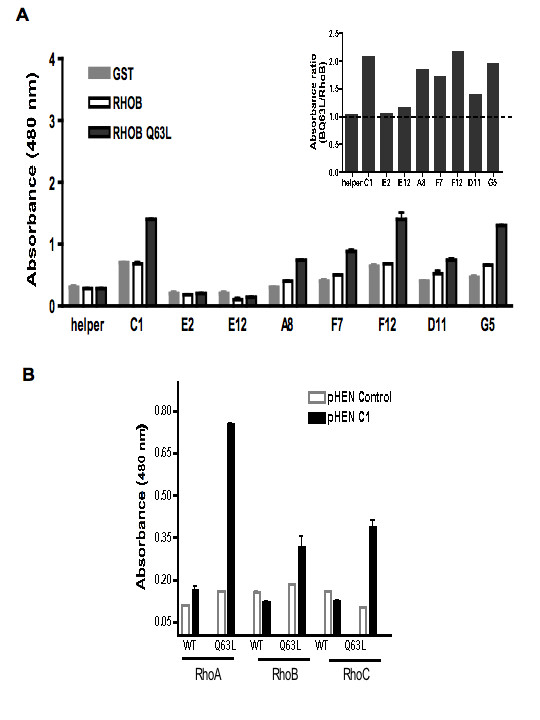
**Phage binding specificity towards RhoQ63L compared to wild-type Rho**. **A: **Ten monoclonal phages from *E. coli *supernatant were analyzed for binding to GST (grey columns), GST-RhoB (white columns) and GST-RhoBQ63L (black columns) protein immobilized on an ELISA plate. Bound phages were detected with horseradish peroxydase-labeled anti-M13 using TMB as substrate. Helper phage was used as a control. Results are expressed as absorbance at 480 nm. **Insert**: ratio of absorbance of binding to RhoBQ63L to absorbance of binding to RhoB. **B**: Selectivity of C1 phages on WT and activated Q63L form of Rho. 10^10 ^clones of C1 (black columns) and control (white columns) phage were analyzed for binding to GST-RhoA, RhoB and RhoC, both wild type (WT) and Q63L forms, immobilized on a glutathione ELISA plate. Bound phages were detected with horseradish peroxydase-labeled anti-M13 using TMB as substrate. Results are expressed as absorbance at 480 nm. Concentrations of GST-Rho proteins in each well were monitored by anti-GST (not shown). The graph is representative for 3 experiments, and each binding assay was performed in duplicate.

To characterize the 8 RhoBQ63L selective phages, their specificity was first examined by analyzing their interaction with GST, GST-RhoB and GST-RhoBQ63L (Fig 2A). Six of the phages showed preferential binding to the GTP-locked RhoBQ63L mutant, while two clones (E2 and E12) failed to interact and were discarded (Fig 2A).

To assess the specificity of the selected scFv against the GTP-bound form of the three Rho proteins of interest, C1 and control phages were tested by ELISA with the wild type form of RhoA, B and C (WT) and their corresponding active forms (Q63L). As shown in Fig 2B, C1 phage showed significant reactivity against the Q63L mutant of all three Rho proteins. In contrast, the reactivity against the WT form was very low in most cases, and within the range of the background response. As predicted, the C1 phage does not discriminate between the GTP-bound form of RhoA, B, C, suggesting that C1 is directed against the GTP binding domain or against the effector-binding domain shared at almost 100% identity among the three proteins. This also suggests that the carboxy terminal hyper-variable domain of these proteins, which functions to target membrane localization, has little importance in the conformation of the active form.

**Figure 3:**
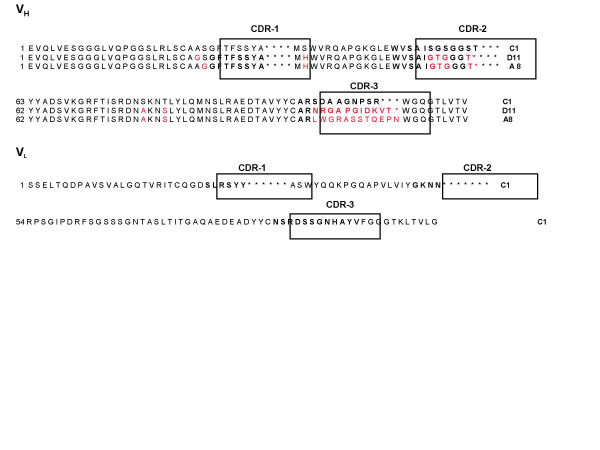
**Amino-acid sequence analysis of the identified scFvs specific for RhoBQ63L binding**. Amino-acid sequence alignments of the VH and VL regions of the scFvs C1, D11 and A8 compared to the human immunoglobulins sequences (imgt). Sequences highlighted in red vary from the amino-acid sequence of scFv C1. Underlined sequences correspond to CDR. Asterisks indicate the missing amino-acid that would generate the usual CDR.

DNA sequencing of the 6 preselected clones revealed three unique scFv sequences for phages C1, D11 and A8 (Fig 3), although scFv D11 and A8 only contained sequence for the variable heavy chain, V_H_. The major differences between the amino-acid sequences of the three clones were concentrated in the hypervariable complementary-determining regions, CDR2 and CDR3, of the V_H_. The A8 and D11 phages proved to be unstable. They quickly lost their recognition properties, and were discarded. Thus, only the C1 phage was kept for further analysis.

### Biochemical characterization of the specific binding of scFv C1

**Figure 4:**
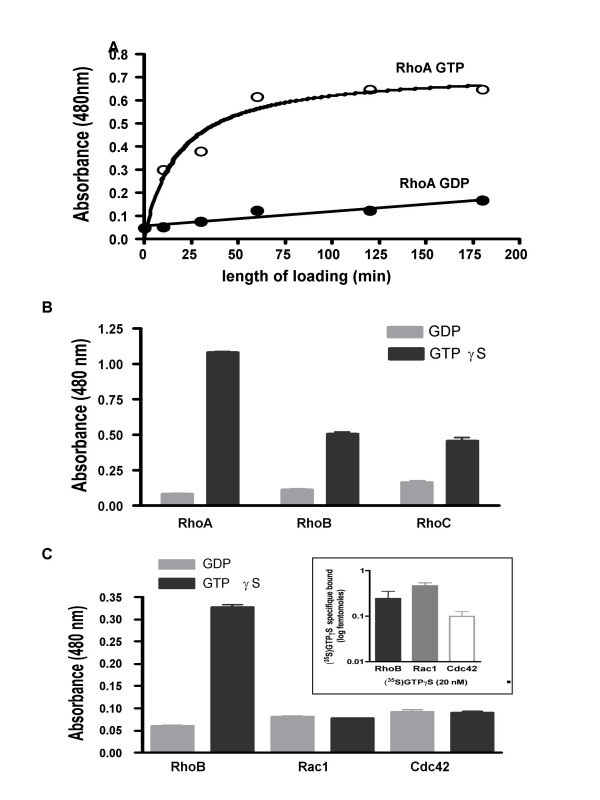
**Specificity of C1 phage binding to GTP-bound Rho family members**. **A**: Kinetics of GTP loading on RhoA. GST-RhoA immobilized on gluthatione beads was loaded with 100 μM of GDP and GTPγS at 37°C. 10^11^clones of C1 phages were incubated with loaded (GTP or GDP) GST-RhoA-bound beads. Bound phages were detected with horseradish peroxydase-labeled anti-M13 using TMB as substrate. Results are expressed as absorbance at 480 nm. The graph is representative of 2 independents experiments. GST-RhoA, B and C (**B**) and GST-RhoB, Rac1 and Cdc42 (**C**) were loaded with 200 μM of GDP or GTPγS for 30 min and purified on glutathione ELISA plates. 10^10 ^clones of C1 phages were incubated in each well. Bound phages were detected with horseradish peroxydase-labeled anti-M13 using TMB as substrate. Results are expressed as absorbance at 480 nm. Amount of GST protein was quantified with goat anti-GST antibody followed by horseradish peroxydase-labeled anti-goat (not shown). The graph is representative of 3 experiments, each binding assay performed in duplicate. **Insert**: Specific radioactivity binding of [^35^S] GTPγS on RhoB, Rac1 and Cdc42. GST-RhoB, Rac1 and Cdc42 were loaded with 20 nM [^35^S] GTPγS in the presence (non specific binding) or not (total binding) of 200 μM unlabeled GTP for 30 minutes at 37°C and purified on gluthatione ELISA plates. Radioactivity was measured in each well. The difference between the total binding and the non specific binding represent the specific binding. The graph is representative of 2 experiments, each performed in triplicate.

In order to further determine whether C1 specifically detected Rho proteins in their GTP-bound conformation, we next assayed C1 binding to Rho protein preloaded with GTP. First, GST-RhoA expressed and purified onto glutathione beads was loaded with GDP or the slowly hydrolyzable analogue of GTP, GTPγS, to ensure maximum loading of RhoA with the nucleotide [25]. C1 phage was next added and incubated for 1 hour, and bound C1 phages were measured with anti-M13. As seen in Fig 4A, the C1 binding increased up to 75 min after loading with GTPγS until a steady state was reached. Under similar incubation conditions, in the presence of GDP, the C1 phage recognition of RhoA did not change. To further confirm the Rho GTP-bound conformational specificity of C1, nucleotide-binding assays with RhoB and RhoC were tested in comparison with RhoA. C1 phages bound specifically to GTPγS but not GDP preloaded RhoA, RhoB and RhoC (Fig 4B).

Interestingly, when we tested the binding of C1 phage against the GTP-bound form of Rac1 and Cdc42, two well-characterized members of two separate subfamilies of Rho GTPases, C1 phages bound selectively on GTPγS-RhoB but not GTPγS-Rac1 or GTPγS-Cdc42 (Fig 4C), despite their ability to successfully bind GTP (insert Fig 4C). Together this demonstrated that the scFv C1-pIII fusion protein specifically targeted the GTP-bound form of RhoA, RhoB and RhoC, and was unable to recognize GTPγS or GDP preloaded Rac1 and Cdc42, two other Rho subfamily members.

### Expression and activity analysis of recombinant scFvC1

**Figure 5:**
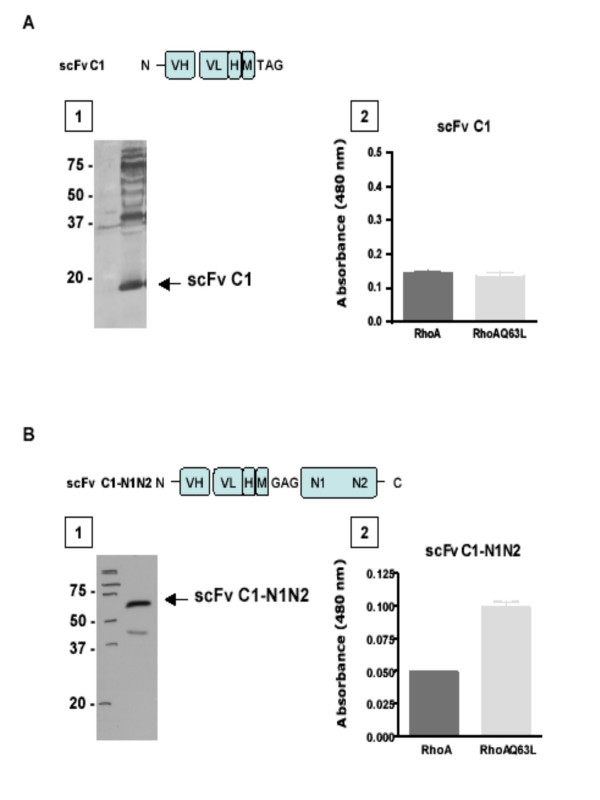
**Binding activity of scFv C1 compared to scFv C1-N1N2**. (**A**) scFv C1 and (**B) **scFv C1-N1N2 crude extracts analyzed by (1) Western Blot and (2) ELISA. (1) scFvs were resolved on 12,5% SDS-PAGE, immunoblotted with c-myc antibody, and visualized by enhanced chemiluminescence as described in Methods. (2) scFvs from crude extract were analysed for binding to GST-RhoA and GST-RhoAQ63L protein immobilized on an ELISA plate. Bound scFvs were detected with horseradish peroxydase-labeled anti-c-myc using TMB as substrate. Results are expressed as absorbance at 480 nm. Graphs are representative of 6 experiments, each performed in duplicate.

To further characterize scFv C1 binding, we constructed and expressed recombinant scFv C1 protein. The pHEN2 phagemid vector carries an amber codon (TAG) at the C-terminal of the scFv sequence and prior to the M13 pIII sequence (Fig 1B, top panel). Non-suppressive strains, such as BL21, interpret the amber codon as a translation stop signal, thus the scFv C1 is expressed in soluble form and can be purified via the histidine tags. Crude bacterial extract from cells expressing scFv C1, as confirmed by Western Blot analysis with anti-c-myc antibodies, lost its ability to bind RhoAQ63L (Fig 5A). The multiple supplementary bands observed in the blot would be explained by the renowned propensity of scFv to form dimeric and higher-order aggregates. This loss of function is a common observation when phage-display derived scFvs are expressed as soluble scFvs. Jensen *et al*. reported that an inactive antibody fragment can be functionally rescued by fusion to the N-terminal domain of the original phage display fusion partner, filamentous phage protein III [26]. We employed a similar approach and mutated the amber codon to allow translation and fusion to the N terminal of pIII, as well as removed the C terminal of pIII (Fig 1B, scFv C1-N1N2, bottom panel). As shown in Fig 5B, scFv C1-N1N2 exhibited the predicted molecular mass of 60 kDa. Furthermore, the bacterial lysate expressing scFv C1-N1N2 was now able to selectively recognize RhoAQ63L versus RhoA as determined by ELISA. Competitive experiments between scFvC1-N1N2 and the Rho binding domain of Rhotekin (RBD) have been analyzed and are summarized in Table 1. RBD alone is not stable enough to be used in this study, however the chimere GST-RBD is able to compete with scFvC1-N1N2 on RhoAQ63L and shows an affinity more than 10 fold higher than the scFv C1-N1N2. Affinities for GDP-Rho, GTP-Cdc42 and GTP-Rac1 are too weak to be determined.

**Table 1:** ELISA competition studies on RhoAQ63L coated plate. ScFvC1-N1N2 (0.4 μM) is competed by increasing concentrations of GST-RBD, GDP bound RhoA, GTP bound Cdc 42, GTP bound Rac 1. K_D _of GST-RBD was also determined by surface plasmon resonance on GST-RhoAQ63L.

	GTP-RhoA	GDP-RhoA	GTP-Cdc42	GTP-Rac1
GST-RBD KD (μM)	0.2 ± 0.2	> 50	> 50	> 50
ScFvC1N1N2 KD (μM)	3 ± 1	> 50	> 50	> 50

**Figure 6:**
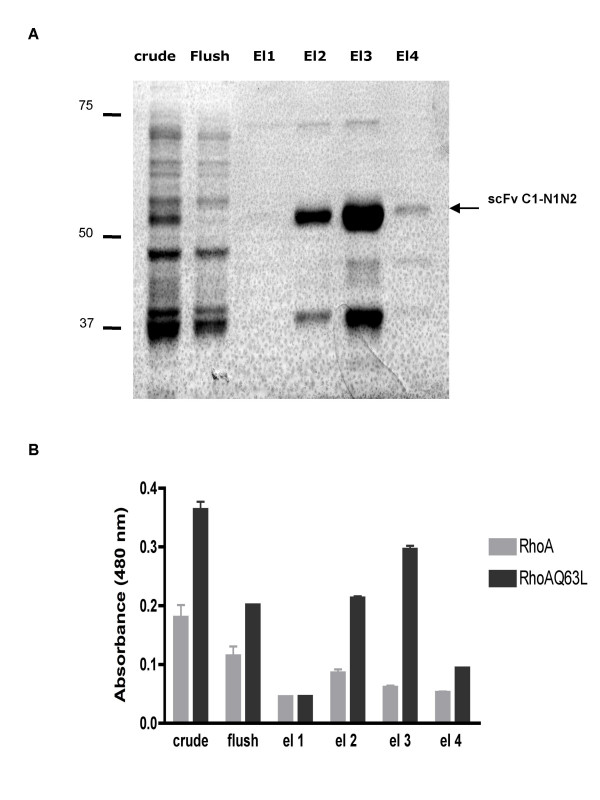
**Characterization of enriched scFv C1-N1N2**. **A: **scFv from crude extract was purified on Ni-NTA beads. Fractions (20 μl) were resolved on a 12.5% SDS-PAGE gel, and visualized by Coomassie Blue staining. *Crude*: total extract; *flush*: non bound scFv; El (1–4): elution fractions 1 to 4. **B**: Each step of purification (1:10 diluted elutions in 3% milk-PBS) was analyzed for binding to GST-RhoA and GST-RhoAQ63L protein immobilized on an ELISA plate. Bound scFv were detected with horseradish peroxydase-labeled anti-myc using TMB as substrate. Results are expressed as absorbance at 480 nm. Graphs are representative of more than 5 experiments, each performed in duplicate.

The new recombinant C1-N1N2 fusion protein was expressed more easily in a soluble form, and showed higher binding to RhoAQ63L. We then purified scFv C1-N1N2 from crude extract by incubation with Ni-NTA agarose beads and elution with imidazole, and aliquots from each step were analyzed by SDS PAGE (Fig 6A). The binding activity towards RhoAQ63L of scFv C1-N1N2 crude extract and the four elutions fractions were tested and confirmed by ELISA (Fig 6B). As seen on the SDS PAGE various proteins with lower molecular mass than scFvC1-N1N2 are co-purified with the desired probe. As reported by others these proteins would be the result of translational arrest in the N1N2 sequence of the phage PIII.

To measure the affinity of this scFv, surface plasmon resonance (SPR) experiments were performed on a Biacore 3000 instrument [27]. Using the previously described methodology by Horn *et al*, we bound GST-RhoA loaded with GDP on one flowcell (FC1), and GST-RhoA loaded with GTPγS on a second flowcell (FC2) [28]. C1-N1N2 "antibody" was injected at a concentration of 2.5 and 8 μM using a flow rate of 20 μl/min. In order to test the specificity of scFv C1-N1N2 for the GTP-bound form of RhoA, differential responses (FC2-FC1) were recorded and analyzed using the Biaevaluation 4.0 software (Biacore AB). The results supported the GTP-bound conformational specificity, with an apparent dissociation constant (K_D_) in the range of 3 ± 1 μM (data not shown). Similarly, affinity of a scFv molecule for GTP-bound Ras was previously reported in the range of 1.3 μM [29]. Same results were found with GST-Cdc 42 and GST-Rac 1 loaded with GTPγS are coated on FC1 in front of GST-RhoA loaded with GTPγS on FC2.

### Recombinant scFv C1-N1N2 used as sensor of GTP-bound Rho *in vitro *and *in vivo*

**Figure 7:**
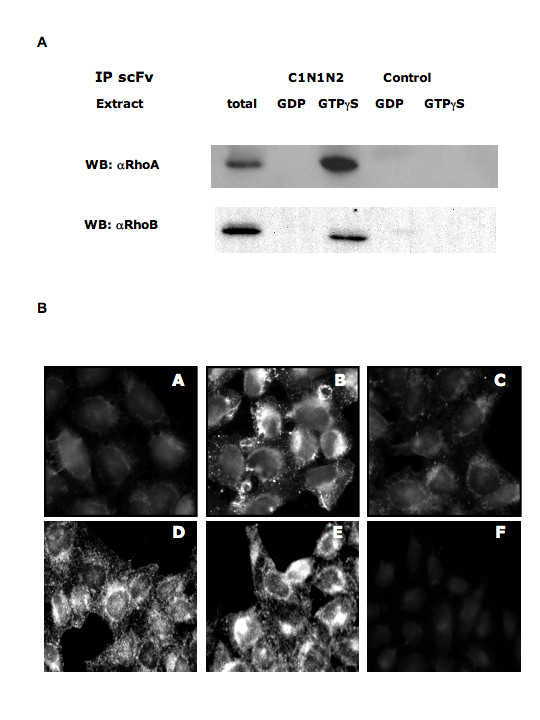
**scFv C1-N1N2 specifically recognizes GTP-bound Rho *in vitro *and *in vivo***. **A: **scFv C1-N1N2 immunoprecipitation (IP) of GTP-bound RhoA and RhoB. Crude bacterial lysates of recombinant RhoA and RhoB loaded with either GTP or GDP were incubated with **scFv **C1-N1N2 fixed on Ni-beads. An irrevelant scFv was used as control. Complexes on beads were resolved by SDS-PAGE and immunobloted with anti-RhoA and anti-RhoB. Western Blot is representative of 2 independent experiments. **B: **Immunofluorescence shows that scFv C1-N1N2 specifically binds to activated HeLa cells. Suspension containing scFv C1-N1N2 was incubated with GDP-loaded RhoA(**A **and **B**) or GTPγS-preloaded RhoA beads (**C**). Twenty-four hours after seeding, HeLa cells were serum-starved for 48 h and activated with 10% SVF and EGF (100 ng/ml) for 1 hour. Cells were fixed, permabilized and incubated with supernatants from scFv C1-N1N2-Rho incubation and anti-c-myc FITC conjugate secondary antibody. **(A**) Non-activated HeLa cells incubated with the antibody scFv C1-N1N2 preincubated with GDP-loaded RhoA beads, **(B**) EGF-activated HeLa cells incubated with the antibody scFv C1-N1N2 preincubated with GDP-loaded RhoA beads **(C**) EGF-activated HeLa cells incubated with the antibody scFv C1-N1N2 preincubated with GTPγS-loaded RhoA beads. (**D**) Non-activated HeLa cells incubated with the commercial Rhoa antibody, (**E**) EGF-activated HeLa cells incubated with the commercial RhoA antibody, (**F**) EGF-activated HeLa cells incubated with irrelevant scFv (anti-tyroglobulin). Pictures are representative of 2 independent experiments.

We determined the ability of the antibody to immunoprecipitate RhoA and RhoB in their GTP-bound conformations. Crude bacterial extracts expressing scFv C1-N1N2 was incubated with Ni-NTA beads to immobilize the antibody, and bacterial lysate containing recombinant RhoA or RhoB preloaded with GDP or GTP were mixed with these beads. RhoA and RhoB specifically bound to the scFv C1-N1N2 beads were analysed by Western Blot with commercial anti-RhoA and anti-RhoB antibodies (Fig 7A). The scFv C1-N1N2 on Ni-NTA beads selectively associated with the GTP-bound form of both RhoA and RhoB, confirming the specificity of this new antibody.

The ability of scFv C1-N1N2 to identify activated Rho was next tested *in vivo *on activated HeLa cells by immunofluorescence. After serum deprivation (48 h), HeLa cells were activated 1 hour with EGF (100 ng/ml) and serum (FBS 10%) and immunostained with scFvC1-N1N2, irrelevant scFv (anti-thyroglobulin) and anti-c-myc antibodies. We observed that staining with scFv C1-N1N2 (Fig. 7B panel A andB), but not the irrelevant scFv (Fig. 7B panelF), produced a positive immunofluoresence signal specifically in activated cells (Panel B). To confirm the signal specificity, prior to staining, scFv C1-N1N2 was first incubated with GTPγS- preloaded RhoA to exhauste the selective scFv (negative control) or GDP-preloaded RhoA which would have any effect on scFv activity (positive control). On serum starved cells, little signal was detected with either the scFv C1-N1N2 antibody preincubated with GDP-bound RhoA (Fig 7B panel A) or GTPγS-bound RhoA (not shown), although Rho A is detected by a commercial antibody (Fig 7B panelD). Upon activation, a signal was visualized with the scFv C1-N1N2 preincubated with GDP-bound RhoA (Fig 7B panel B), showing labelling of the plasma membrane and punctate cytoplasmic structures consistent with previous reports for Rho localization [30]. Similar labelling is obtained with commercial anti-RhoA (Fig 7B panel E). Moreover, this signal was suppressed by preincubation of the scFv C1-N1N2 antibody with GTPγS-preloaded RhoA (Fig 7B panel C). This data strongly suggests that scFv C1-N1N2 is able to recognize endogenous activated and GTP-bound Rho at the cellular level.

## Conclusion

Binding domains of effector proteins have proven to be useful as conformational sensors in analyzing the spatiotemporal activation of GTPases [31,32]. In this study, we have shown that a phage display approach identified a specific phage for the GTP-bound form of RhoA, B and C. The expressed and purified scFvC1-N1N2 protein proceeded from this phage is able to recognize the three Rho proteins (A, B and C) loaded with GTPγS with great specificity, and detect activation of endogenous Rho protein in fixed cells. This finding implicates the potential for scFv C1-N1N2 as a useful tool to compare the level of activated Rho in cancer tissues versus normal tissues.

## Methods

### Construction of pHEN vectors

The schematic for vector construction is presented in Figure 1. Quick-Change (Stratagene) was used to remove the amber stop codon between scFv and protein III genes in pHEN 2, using the following primers: 5'CTGAATGGGGCCGCAGAGACTG TTGAAAGTTG 3' and 5'CAACTTTCAACAGTCTCTGCGGCCCCATTCAG 3'. A new EcoRI site was introduced downstream of the pIII N2 region by PCR amplification of this modified plasmid (pHEN C1-pIII) using primers LMB3, 5'-CAGGAAACAGCTATGAC-3', and N2 (EcoR1), 5'CCGGA ATTCGCCGCCGCCAGCATTGAC 3'. The final pHEN C1-N1N2 vector was constructed by cloning the NcoI/EcoRI digested PCR product into the NcoI and EcoRI digested pHEN C1-pIII plasmid. Constructs were verified by sequencing.

### GST-fusion vectors and protein expression

The Q63L mutation of RhoA, RhoB, and RhoC was generated in the original wild-type cDNA of RhoA, RhoB, RhoC (a gift from Dr. P. Sheffield [33]) cloned into the pGSTparallel2 vector, in frame at the 3' of glutathione-S-transferase, using the QuickChange Site-Directed Mutagenesis kit (Stratagene). The primer for RhoAQ63L 5' GGACACAGCTGGGC**TC**GAGGATTATGATCG 3', for RhoBQ63L, 5' GGACACGGCGGGCC**TC**GAGGACTACGACCG 3' and for RhoCQ63L 5' GGACACAGCAGGGC**TC**GAGGACTATGATCG 3', introduce an internal XhoI restriction site in the pGSTparallel2 DNA sequence. pGEX plasmids expressing GST-Cdc42 and GST-Rac1 were kindly provided by Dr. A. Blangy (CRBM-CNRS, France).

Recombinant GST fusion Rho proteins were expressed and purified in a protease deficient strain (*E. coli *BL21). Bacteria were grown at 37°C in 1 liter of LB medium containing 100 μg/ml ampicillin until an OD_600 _= 0.4 was reached. Protein expression of RhoA, RhoB, RhoC and Rac was induced by overnight incubation (3 hours for Cdc42) with 0.1 mM of isopropyl-β-D-glactopyranoside (IPTG) at 20°C, and cells were harvested. Bacteria pellets were frozen at -20°C for 15–20 minutes, and subsequently resuspended at 4°C in 50 mM Tris-HCl, pH 7.5, 150 mM NaCl, 5 mM MgCl_2 _(PBS, 2 mM MgCl_2 _for Cdc42) at 1:100 or 1:10 of the original culture volume. Cells were incubated on ice for 30 minutes, sonicated for three 10 sec rounds, and lysate was centrifuged at 7,000 × g for 30 minutes. The supernatant was purified by Reacti-Bind™ glutathione coated strip-well plates (Pierce) or by glutathione sepharose beads (Amersham Pharmacia Biotech) according to the manufacturer's instructions.

### Phage libraries and antibody phage display screening

The Griffin.1 library [24], a human synthetic V_H_+V_L _scFv phage library in a phagemid vector, and the positive control *Escherichia coli *TG1 (TG1 containing an anti-tyroglobulin clone) were generously provided by Fiona Sait of The Medical Research Council (Cambridge, England). We followed the protocol provided with the Griffin.1 library [34], as previously described [35]

Briefly, for the first round of selection, phages were incubated with GST-RhoBQ63L-bound glutathione beads (10 μg of Rho protein). For the subsequent three rounds, bound unspecific phages were removed by first incubation with GST-beads, and unbound phages were incubated with GST-RhoBQ63L beads, then eluted and amplified. For the last round, the phages bound on GST-RhoBQ63L beads were competed with soluble wild type recombinant RhoB (80 μg).

Reactivity of the identified phages was tested with ELISA and positive clones were sequenced. Phagemids DNAs from individuals colonies were extracted and purified with the Quiafilter plasmid purification kit (Qiagen) and sequenced with the primers LMB3 5'-CAGGAAACAGCTATGAC-3' and pHENseq 5'-CTATGCGGCCCCATTCA-3'. The sequences are compared to the human immunoglobulins sequences and aligned with FR-IMGT and CDR-IMGT delimitation [36].

### Monoclonal phage ELISA

Briefly, recombinant GST-Rho proteins were incubated on Reacti-Bind™ glutathione coated plates for 1 hour at room temperature. One group of 48 colonies and another of 96 colonies from the fifth round of selection were picked manually and tested by ELISA as previously described [37].

### Kinetics of Rho GTP loading

Recombinant GST-RhoA purified on glutathione beads was loaded with 0.1 mM of GDP or GTPγS in PBS 10 mM EDTA at 37°C. Loading was repeated several times. A suspension of C1 phages (10^11 ^clones) in 3% MPBS, 10 mM MgCl_2 _was incubated with loaded GST-RhoA beads for 1 hour at room temperature. Beads were washed 3 times with 0.1% Triton ×100 in PBS. A suspension of peroxidase-labeled anti-M13 antibody (1:5000 dilution in 2% milk in PBS) was added for 1 hour and the reaction was assayed, using 3,3',5,5' tetramethylbenzidine substrate (Sigma). The reaction was stopped by adding 1 M sulfuric acid, and the absorbance was read at 480 nm.

### Production and purification of soluble single-chain Fv antibody

BL21 *E coli *non-suppressor strain was transformed with the selected pHEN plasmid, and a selected colony was grown in 100 mL of 2xTY medium containing 100 μg/mL ampicillin and 2% glucose at 37°C. At proper concentration (OD_600 _= 0.8), the culture was centrifuged at 3,300 × g for 10 minutes at 4°C. Bacteria were resuspended in 2xTY medium containing 100 μg/mL ampicillin and induced with 0.1 mM IPTG overnight at 30°C (no more than 16 h). Bacteria were harvested, resuspended in spheroplast buffer (200 mM Tris-HCl, pH 8, 0.5 mM EDTA, 0.5 M sucrose) at 1:10 of the culture volume and incubated on ice for 30 minutes. The sample was centrifuged at 3,300 × g for 10 minutes, and the supernatant containing the scFv was directly subjected to ELISA analysis.

For scFvs purification, bacteria were harvested, resuspended in 50 mM Tris-HCl, pH 7.5, 150 mM NaCl, 2.5 mM MgCl (1/10 of the culture volume), incubated on ice for 30 minutes and sonicated five rounds for 10 sec. The cell lysate was centrifuged at 3,300 × g for 10 minutes, and the supernatant was centrifuged at 17,500 × g for 1 hour. The supernatant containing the scFvs was adjusted to 300 mM NaCl and 1 mM imidazole, and the scFvs were concentrated on 1 ml of 50% slurry Ni-NTA agarose (Qiagen) at 4°C with shaking for at least two hours. After washing with 50 mM Tris-HCl, pH 7.5, 300 mM NaCl, 10 mM imidazole, bound scFvs were eluted with 50 mM Tris-HCl, pH 7.5, 300 mM NaCl, 250 mM imidazole. Samples of all fractions were analysed by SDS-PAGE. ScFvs-containing fractions were pooled and applied to a PD-10 column (G25M Sephadex, GE Healthcare) equilibrated with 50 mM Tris-HCl, pH 7.5. Desalted scFvs were analyzed by SDS-PAGE and Coomassie blue staining or Western blotting with anti-c-myc. All fractions containing scFvs were tested by ELISA in conditions detailed above.

### Antibodies

Mouse anti-RhoA (26-C4) and rabbit anti-RhoB (sc-180) antibodies were obtained from Santa Cruz Technology (Santa Cruz, CA); goat anti-GST, mouse anti-M13 (HRP) antibodies were obtained from Amersham Pharmacia Biotech (AB, Les Ulis, France); goat anti-c-myc (HRP) antibodies were obtained from Interchim (Montluçon, France), mouse anti-c-myc-FITC conjugate antibodies were obtained from Zymed (Invitrogen, Cergy Pontoise, France).

### Immunoprecipitation-Western Blot analysis

Crude protein lysates containing recombinant RhoA and RhoB were loaded with 2 mM GDP or 0.2 mM GTPγS in PBS, 10 mM EDTA for 30 min at 30°C. For immunoprecipitation, beads bound by scFv C1-N1N2 or scFv control (anti-tyroglobulin) antibodies were incubated with loaded Rho protein suspension for 45 min at 4°C. Beads were washed 3 times with 50 mM Tris-HCl, pH 7.5, 150 mM NaCl, 30 mM MgCl_2_, 0.1% Triton ×100. To remove scFv-protein complexes, beads were denatured in 2X Laemmli reducing sample buffer, boiled for 5 minutes and separated on 12.5% SDS-PAGE for Western Blot analysis with anti-RhoA or anti-RhoB antibodies followed by HRP-conjugated secondary antibodies. Protein detection was performed using the enhanced and chemiluminescence (ECL) system.

### Guanine nucleotide-binding assay

Recombinant GST-RhoB, GST-Rac1 and GST-Cdc42 were preloaded with [^35^S] GTPγS (1000 Ci/mmol, 1 mCi/mL, GE Healthcare) or with [^3^H] GDP (12 Ci/mmol, 1 mCi/mL, GE Healthcare) at different concentrations (0.2 to 20 10^-9 ^M) in 10 mM EDTA for 30 min at 37°C. Specific binding was obtained in the presence of 0.2 mM unlabeled nucleotide. Loaded crude extracts were purified on Reacti-Bind™ glutathione coated plates for 1 hour at room temperature. Plates were washed twice with PBS 0.05% Tween-20. 1 N NaOH was added to wells to remove bound GST-Rho, and supernatant was collected and diluted in the cocktail used for counting aqueous samples (Ultima Gold, PerkinElmer). Radioactivity bound to GST-Rho was determined by scintillation counting.

### Immunofluorescence

Recombinant GST-RhoA fixed on beads (about 600 μg of protein) was loaded with 100 μM GTPγS or GDP in 10 mM EDTA for 1 hour at 37°C. Purified scFvs (0.5 mg/ml) was incubated with beads for 1 hour at room temperature, and incubation was repeated twice. HeLa cells (ATCC, CCL-2) grown on coverslips were stimulated with 100 ng/ml of EGF (Sigma) and 10% FBS for one hour, then fixed with paraformaldehyde (Cytofix) and permeabilized with Cytoperm (BD Biosciences). Supernatant alone or containing scFvs after incubation with loaded beads was incubated with cells for 90 minutes. Coverslips were rinsed once in PBS, 5 mM MgCl_2 _and incubated with 2 μg/ml of anti-c-myc FITC conjugate secondary antibody (9E10, Zymed). Staining was visualized using a Nikon eclipse 90i microscope equipped with fluorescence FITC filters (Semrock) and a CoolsnapHQ^2 ^camera (Ropper). Images were acquired using NIS-Element Ar.

## Abbreviations

scFv: single chain Fragment variable; GST: glutathione sulfonyl transferase; MPBS:milk phosphate buffer saline; EGF:epidermal growth factor; FBS: foetal bovine serum; FITC: fluorescein isothiocyanate; RBD: Rho binding domain of Rhotekin

## Authors' contributions

MG constructed the plasmids, generated recombinant proteins for analysis, performed the ELISA, pull down and immunofluorescence studies, and participated in drafting the manuscript. PC generated the *E. coli *phage display and contributed to the production and the biochemical characterization of scFvC1. ILM contributed to the plasmid and GST-proteins constructions, as well as drafted the manuscript. CMG assisted with the radionucleotide assay. GF designed and coordinated the study, helped to draft the manuscript, and organized funding. JCF drafted the manuscript and participated in the design and coordination of the study. All authors read and approved the final manuscript.
